# Red cell distribution width and associated factors among hypertensive patients attending Arba Minch General Hospital, Southern Ethiopia: A comparative cross-sectional study

**DOI:** 10.1371/journal.pone.0336409

**Published:** 2025-11-13

**Authors:** Orgeta Robsha, Selamu Girma, Teklu Teshome, Tesfaye Kanko

**Affiliations:** 1 Department of Biomedical Sciences, School of Medicine, College of Medicine and Health Sciences, Dilla University, Dilla, Ethiopia; 2 Department of Biomedical Sciences, School of Medicine, College of Medicine and Health Sciences, Arba Minch University, Arba Minch, Ethiopia; University of Montenegro-Faculty of Medicine, MONTENEGRO

## Abstract

**Background:**

Red cell distribution width (RDW) has been increasingly linked with cardiovascular and metabolic conditions. However, its clinical relevance in hypertension remains underexplored in Ethiopia. This study aimed to assess RDW levels, associated factors, and its potential utility in hypertension management.

**Methods:**

An institution-based comparative cross-sectional study was conducted from November 2023 to January 2024 among 70 hypertensive patients and 70 age- and sex-matched normotensive controls. Hypertensive participants were consecutively recruited from the hypertension outpatient clinic, while controls were recruited from the general outpatient department using systematic random sampling. Data were collected using a pretested and structured interviewer-administered questionnaire via the KOBO Collect to obtain sociodemographic, lifestyle, and clinical information. Five milliliters of venous blood were drawn from each participant for hematological and biochemical analyses. Data were entered and analyzed using SPSS version 26.0. Independent t-test and Mann–Whitney U-test were used for group comparisons, multivariable logistic regression assessed associations, and Spearman correlation tested RDW versus hypertension duration. Receiver operating characteristic (ROC) curve analysis was performed to determine the predictive ability of RDW for detecting disease severity. A p-value < 0.05 was considered statistically significant.

**Results:**

Hypertensive patients had significantly higher mean RDW values compared to normotensive controls (15.1 ± 2.0% vs. 13.0 ± 2.0%, p < 0.001). RDW was higher in hypertensive patients with stage II (16.35 ± 2.60%) and poorly controlled blood pressure (16.00 ± 2.10%) compared to stage I (14.95 ± 2.10%) and controlled blood pressure (14.70 ± 1.20%). RDW showed a positive but non-significant correlation with hypertension duration (rs = 0.127, P = 0.295) and demonstrated a predictive power of 74.1% for detecting hypertension severity at a cut-off value of 14.5%. RDW was significantly elevated among participants who consumed alcohol (AOR = 6.48; 95% CI: 1.92–21.85), chewed khat (AOR = 5.65; 95% CI: 1.06–29.9), or had elevated C-reactive protein (AOR = 5.90; 95% CI: 1.67–20.89) and serum creatinine (AOR = 4.35; 95% CI: 1.19–15.91).

**Conclusions:**

A higher RDW reported among hypertensive patients compared to their counterparts, with RDW increasing across hypertension stages. These findings suggest a possible role of RDW as an indicator of hypertension progression. Therefore, assessing RDW may provide supportive information for the early identification and management of hypertension-related complications.

## Introduction

Hypertension is defined as systolic blood pressure (SBP) ≥140 mmHg and/or diastolic blood pressure (DBP) ≥90 mmHg [1]. Its prevalence increases with age and is particularly common among older adults [2]. Globally, hypertension affects approximately 31% of the adult population [3], with a higher burden in low- and middle-income countries (31.5%) compared with high-income countries (28.5%) [4]. Although several risk factors have been identified, about 90% of essential hypertension cases have no clearly defined etiology, while only 10% result from identifiable and potentially reversible secondary causes [5,6]. Because it is often asymptomatic, hypertension is frequently referred to as a “silent killer” [7].

In 2022, an estimated 1.13 billion people had hypertension worldwide, projected to rise to 1.56 billion by 2025 (up by 30%) [8]. Hypertension causes approximately 9.4 million deaths annually and accounts for 7% of the global disease burden in disability-adjusted life years [9]. It is a major risk factor for stroke, congestive heart failure, myocardial infarction, chronic kidney disease, and other cardiovascular complications [10–12]. In Africa, prevalence is estimated at 30.8%, with a growing impact on rural communities, low-income households, and younger populations [13]. This increase is largely attributed to population aging, rapid urbanization, dietary changes, and reduced physical activity [14]. In Sub-Saharan Africa, prevalence rates reach up to 38%, and between 44% and 93% of hypertensive adults are unaware of their condition [15,16]

In Ethiopia, prevalence ranges from 7.7% to 41.9%, with a pooled national estimate of 19.6% [17,18]. Hypertension is the seventh leading cause of mortality, accounting for 1.4% of all deaths, and prevalence in the current study area has been reported at 35.2% [19].

Red cell distribution width (RDW) is a parameter of the complete blood count (CBC) that quantifies the variation in erythrocyte size, reflecting anisocytosis, and is routinely used to differentiate types of anemia [20]. RDW is calculated as the ratio of the standard deviation of erythrocyte volume to the mean corpuscular volume, expressed as a percentage [21]. The normal reference range for RDW-CV in humans is 11.5–14.5% [22]. Values below this range are generally not clinically significant, whereas increased values indicate anisocytosis [23].

RDW has more recently gained attention as a potential biomarker for several non-hematological conditions, including cardiovascular and metabolic disorder [24]. Elevated RDW has been associated with increased risk of hypertension, heart failure, stroke, and overall mortality. Moreover, in a large population cohort, each 1% increment in RDW corresponded to significantly higher cardiovascular event and mortality risk, independent of anemia status [25]. Emerging evidence suggests that higher RDW has been significantly associated with elevated blood pressure [26]. Hypertensive patients consistently exhibit increased RDW compared to healthy controls, independent of age, inflammatory state, or anemia [27]. Moreover, RDW is recognized as a significant predictor of cardiovascular outcomes, including coronary artery disease, stroke, and hypertensive heart disease [28], and has been linked to the development of incident hypertension independent of established risk factors [29].

However, findings across studies remain inconsistent, with some reporting positive associations between RDW and hypertension [20,29], while others have shown no association [30,31] or even inverse association [32]. The mechanisms underlying these associations are not yet fully elucidated but are thought to involve chronic inflammation, oxidative stress, impaired erythropoiesis, and nutritional deficiencies, all of which can contribute to anisocytosis and elevated RDW [33]. Early diagnosis and timely intervention are critical for reducing the risks associated with hypertension and its complications [34]. Identifying an affordable screening tool, such as RDW, may facilitate early detection of hypertension and help prevent adverse outcomes. However, further studies are required to validate RDW and support its integration into routine clinical practice.

Assessment of RDW levels in patients with hypertension may serve as an early warning system for physicians to identify individuals at increased risk of adverse events such as cardiovascular disease [20,21]. Routine evaluation of RDW, which is inexpensive and widely available, could facilitate periodic monitoring of patients condition and timely clinical intervention.

In Ethiopia, data on the relationship between RDW and hypertension are limited, and the clinical relevance, prognostic value, and population reference ranges of RDW in hypertensive populations remain poorly defined. Existing studies indicate that hypertensive patients and those with chronic conditions such as end-stage renal disease exhibit elevated RDW, suggesting its potential role in cardiovascular risk stratification [35,36]. Given its low cost, accessibility, and routine measurement in hematological testing, evaluating RDW in Ethiopian hypertensive populations could provide valuable insights for early detection, risk assessment, and management strategies. This study aimed to assess RDW levels, identify associated factors, and explore its potential utility in managing hypertension among patients at Arba Minch General Hospital, Southern Ethiopia.

## Materials and methods

### Study setting, design and population

**Fig 1:**
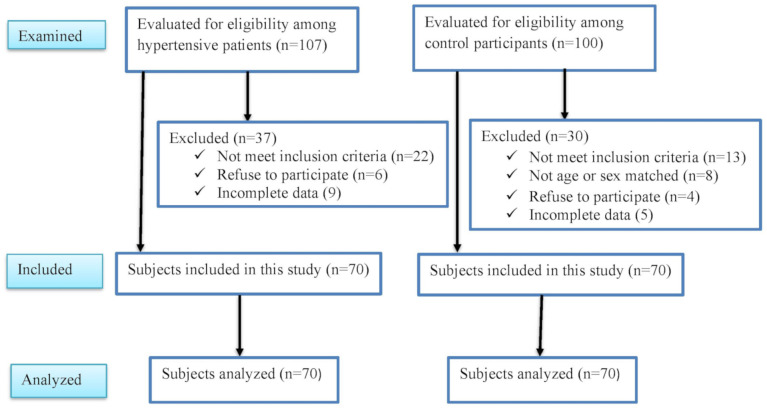
Research flowchart illustrating participant recruitment process.

A comparative cross-sectional study was conducted from November 2023 to January 2024 at Arba Minch General hospital, located in Arba Minch town, approximately 505 km from Addis Ababa, the capital city of Ethiopia. The hospital is the main public hospital in the area, serving a catchment population of more than 2 million people. A total of 140 participants, comprising 70 hypertensive and 70 normotensive controls aged 18–60 years [37], and consent to participate, were included. Hypertensive and normotensive group were defined and classified according to the criteria outlined in the 2018 ESC/ESH Guidelines for the management of arterial hypertension [1]. Hypertensive and normotensive participants were exact sex-matched and nearest age-matched. Participants with suspected hematological disorders, pregnancy, or a history of renal disease, diabetes mellitus, liver disease, peripheral artery disease, chronic heart failure, hematologic malignancy, or blood transfusion within the past three months were excluded. Our study analyzed 140 participants with complete data as illustrated in Fig 1, demonstrating participant recruitment process.

### Sample size determination

The sample size was calculated using G* power (version 3.1) software for two independent means, based on the following assumptions: significance level (α) =5%, effect size (d) = 0.42, and power of 80% (1-β = 0.8). Mean (±SD) red cell distribution width values for hypertensive patients (13.65 ± 1.5) and control subjects (13.15 ± 0.8) were obtained from a previous study conducted in northeast Ethiopia [38]. After adjusting for the calculated sample size, a total of 140 participants were enrolled, comprising 70 hypertensive patients and 70 normotensive controls.

### Sampling procedure and subject selection

The study included individuals with a confirmed diagnosis of hypertension at the hypertension clinic of Arba Minch General Hospital. Hypertensive participants were recruited consecutively from this outpatient clinic, while controls were selected from the general outpatient department using systematic random sampling and confirmed as non-hypertensive by a physician. Written informed consent was obtained from all participants, and data on sociodemographic and behavioral characteristics were collected through face-to-face interviews using a structured questionnaire. Participants who fulfilled the inclusion criteria and were willing to participate were included in the study, ensuring a complete and representative sample of cases and controls.

### Data collection tool and procedure

After obtaining written informed consent, socio-demographic data, clinical history, and behavioral factors were collected by using a pretested, structured, interviewer-administered questionnaire by experienced health care professionals using KOBO collect survey tools. The questionnaire was adapted from previous related studies [36,39,40]. Anthropometric measurements—including weight, height, waist, and hip circumferences—were taken using a digital weighing scale and a standard measuring tape. Body mass index (BMI) and waist-to-hip ratio (WHR) were calculated as weight in kilograms divided by height in meters squared and waist circumference in centimeters to hip circumference in centimeters ratio, respectively. Blood pressure measurement and clinical data were also collected from each participant.

### Sample collection and laboratory analyses

Five milliliters of venous blood were collected from each participant under aseptic conditions by an experienced phlebotomist. Blood samples for hematological analysis, such as red cell distribution width (RDW), were collected into EDTA tubes and analyzed immediately using a Mindray automated hematology analyzer. For biochemical analysis, samples for serum creatinine and C-reactive protein (CRP) were collected in serum separator tubes (SST), allowed to clot for 30 minutes at room temperature, centrifuged at 3,000 rpm for 5 minutes, and the serum was separated and stored at −20°C until analysis. Serum creatinine and CRP levels were measured using a Siemens automated chemistry analyzer. Standard operating procedures were strictly followed throughout collection, handling, transport, and analysis to ensure specimen integrity and reliable results.

### RDW measurement

A 5 mL of venous blood samples were collected from both hypertensive patients and healthy controls by the experienced phlebotomist using ethylene diamine tetraacetic acid (EDTA) tubes and analyzed with a Mindray automated hematology analyzer located in the Hematology Department of Arba Minch General Hospital.

### Blood pressure measurement

Blood pressure (BP) was measured by qualified nurses using a mercury sphygmomanometer and stethoscope, and hypertension was diagnosed based on ESC/ESH guidelines for the management of arterial hypertension [1]. Blood pressure was taken twice, 1–2minutes apart, with additional measurements if the first two readings differed by >10 mmHg, and the average of the last two readings was used.

### Covariates

Our study carefully considered multiple relevant variables that could affect the relationship between RDW and hypertension such as age (years), body mass index (kg/m^2^), smoking status(Yes/No), alcohol use(Yes/No), khat chewing(Yes/No), use of fruit and vegetables(Yes/No), vitamin supplement(Yes/No), meat consumption(Yes/No), salt use(Yes/No), macrocytic drugs use(Yes/No); laboratory test parameters: Red cell distribution width(%), C-reactive protein(mg/dl), serum creatinine(mg/dl) [41–43]. Fruit and vegetable intake, salt consumption, and macrocytic drug use were further detailed in the supplemental questionnaire (S1 File). These covariates were chosen to fully account for potential confounders that could impact the relationship between RDW and hypertension.

### Data quality assurance

In order to manage the data quality, the questionnaire was first developed in English and then translated into Amharic by a qualified medical translator fluent in both languages and then back-translated into English independently by a second qualified translator to ensure accuracy of the tool. Content validity was assessed by three experts in clinical biochemistry and public health. Reliability was evaluated using a pretest on 5% of participants outside the main study population, and internal consistency was assessed using Cronbach’s alpha, with values ≥0.7 considered acceptable. Before the actual data collection commenced, the data collectors and supervisors received one day of intensive training on the study objectives, the questionnaire content, data collection procedures, and ethical issues. In addition, the data collectors underwent practical training on using the KOBO Collect survey tool to record, save, and submit data to the server. Standard operating procedures were strictly followed for sample collection, processing, and transportation; internal quality controls were performed for hematological and biochemical parameter tests to ensure safe procedures, reliable specimens, and accurate results. The investigators and supervisors carefully checked the collected data for completeness before uploading it to the KOBO collect server.

### Statistical analysis

Data were downloaded from the KOBO Collect survey tool, checked for completeness, consistency, and accuracy using Microsoft Excel, and then exported to SPSS version 26.0 for statistical analysis. Descriptive statistics were used to summarize the socio-demographic characteristics, behavioral factors, and clinical history of the study participants. The normality of data distribution was assessed using the Kolmogorov-Smirnov (K-S) test. Normally distributed continuous variables were presented as mean ± standard deviation, and non-normally distributed variables as median with interquartile range. RDW differences between hypertensive cases and normotensive controls were analyzed using an independent t-test or Mann-Whitney U test, as appropriate. Variability of RDW between stages was assessed using Levene’s test for equality of variances. Missing data were not included in the analyses. The correlation between RDW and duration of illness was assessed using Spearman’s correlation. All covariates with a p-value <0.25 in the binary analysis were included in a multivariable logistic regression model. Model fitness was evaluated using the Hosmer-Lemeshow test (p = 0.21). The predictive power of RDW for detecting disease severity was evaluated using Receiver Operating Characteristic (ROC) curve analysis. P-value <0.05 at a 95% confidence level was considered statistically significant. Results were presented in tables, figures, and text.

### Operational definition

Stage I hypertension: a condition in which the systolic blood pressure is 140 mmHg or higher and the diastolic blood pressure is 90 mmHg or higher [44].

Stage II hypertension: a condition in which the systolic blood pressure is 160 mmHg or higher and the diastolic blood pressure is 100 mmHg or higher [44].

Normal RDW-CV: 11.5–14.5%.

Increased RDW: Red cell distribution width >14.5% [45].

Controlled blood pressure: It is systolic blood pressure (SBP) less than 140 mmHg and diastolic blood pressure (DBP) less than 90 mmHg while on antihypertensive medication(s) and/or non-pharmacologic treatment [35].

Uncontrolled blood pressure: It is the SBP more than or equal to 140 mmHg and/or DBP 90 mmHg or higher despite antihypertensive medication [35]

Case: Individuals who have confirmed hypertension and are on follow up.

Control: Non-hypertensive individuals without having diseases or conditions.

### Ethical approval and consent to participate

Ethical clearance was obtained from the Arba Minch University College of Medicine and Health Sciences Institutional Research Ethics Review Board (reference number IRB/230098/2023). An official collaboration letter from the College of Medicine and Health Sciences was submitted to the hospital to secure permission. Written consent was obtained from each participant after providing information about the study’s purpose, objectives, and potential pain or discomfort related to blood sample collection. Confidentiality of information was maintained, and participants were allowed to withdraw at any time. Participants with critically abnormal RDW values had their results communicated to healthcare professionals to facilitate appropriate clinical management.

## Results

### Baseline characteristics of the study participants

A total of 140 study participants, including 70 hypertensive cases and 70 healthy controls, were enrolled in the study. The mean age of hypertensive patients was 39.64 ± 12.51 years (range 20–60), while that of healthy controls was 34.31 ± 9.52 years (range 21–55). In both groups, 41 participants (58.6%) were male. Most hypertensive patients 39(55.7%) and controls 40(57.1%) were urban residents. The majority of study participants 41(58.6%) were married, and 23(32.9%) had completed college or university education.

The mean BMI and waist-to-hip ratio for hypertensive cases were 25.916 ± 2.4 kg/m2 and 0.84 ± 0.07, respectively, compared to 24.41 ± 1.99 kg/m2 and 0.77 ± 0.066 for the control group. Regarding lifestyle factors, majority of patients had not consumed alcohol 40(57.1%) or smoked 48(68.6%), 59(84.3%) reported regular consumption of fruits and vegetables, 47(67.1%) did not consume meat or dairy products, and 42(60%) limited salt intake. Additionally, 59 patients (84.3%) had never used macrocytic drugs, 49(70%) were overweight, and 2(2.9%) were classified as obese.

The mean duration of HTN was 12.57 ± 16.04 months, with 48 patients (68.6%) having hypertension for less than one year. A total of 47 patients (67.1%) reported a family history of HTN. Fifteen patients (21.4%) had previously used traditional medicine, while 4(5.7%) were currently using it. Most patients were classified as stage I hypertension 56(80%) and 44 (62.9%) had uncontrolled blood pressure (Table 1).

**Table 1:** Baseline characteristics of the study participants.

Variable	Hypertensive (n = 70)	Normotensive (n = 70)	P-value
Age (years)*			Chi-squared, 0.014
18–35	30 (42.9%)	40 (57.1%)	
36–55	33 (47.1%)	30 (42.9%)	
> 55	7 (10%)	0 (0%)	
Mean ± SD	39.64 ± 12.51	34.31 ± 9.52	
**Sex**			
Male	41 (58.6%)	41 (58.6%)	Chi-squared, 0.23
Female	29 (41.4%)	29 (41.4%)	Chi-squared, 0.42
**Residence**			
Rural	31 (44.3%)	30 (42.9%)	Chi-squared, 0.37
Urban	39 (55.7%)	40 (57.1%)	Chi-squared, 0.27
**Marital status**			Chi-squared, < 0.01
Single	18 (25.7%)	22 (31.4%)	
Married	41 (58.6%)	41 (58.6%)	
Divorced	4 (5.7%)	2 (2.9%)	
Widowed	7 (10%)	5 (7.1%)	
**Educational status**			Chi-squared, < 0.01
No education	20 (28.6%)	18 (25.7%)	
Primary	11 (15.7%)	7 (10%)	
Secondary	17 (24.3%)	22 (31.4%)	
College/university	22 (31.4%)	23 (32.9%)	
**Occupation**			Chi-squared, < 0.01
Farmer	20 (28.6%)	19 (27.1%)	
Merchant	12 (17.1%)	16 (22.9%)	
Government employee	17 (24.3%)	15 (21.4%)	
Others**	21 (30%)	20 (28.6%)	
**BMI**			Chi-squared, 0.003
Normal weight	19 (27.1%)	38 (54.3%)	
Overweight	49 (70%)	32 (45.7%)	
Obese	2 (2.9%)	0 (0%)	
Mean ± SD	25.92 ± 2.4	24.41 ± 1.99	
**Alcohol use**			Chi-squared, < 0.001
Yes	30 (42.9%)	22 (31.4%)	
No	40 (57.1%)	48 (68.6%)	
**Smoking**			Chi-squared, < 0.001
Yes	22 (31.4%)	5 (7.1%)	
No	48 (68.6%)	65 (92.9%)	
**Khat use**			Chi-squared, < 0.001
Yes	26 (37.1%)	7 (10%)	
No	44 (62.9%)	63 (90%)	
**Fruit/vegetable intake**			Chi-squared, 0.009
Yes	59 (84.3%)	68 (97.1%)	
No	11 (15.7%)	2 (2.9%)	
**Vitamin/nutritional supplement**			Chi-squared, 0.404
Yes	4 (5.7%)	2 (2.9%)	
No	66 (94.3%)	68 (97.1%)	
**Meat/dairy consumption**			Chi-squared, < 0.001
Yes	23 (32.9%)	60 (85.7%)	
No	47 (67.1%)	10 (14.3%)	
**Salt use**			Chi-squared, < 0.001
Yes	28 (40%)	54 (77.1%)	
No	42 (60%)	16 (22.9%)	
**Medication use**			Chi-squared, 0.144
Yes	11 (15.7%)	18 (25.7%)	
No	59 (84.3%)	52 (74.3%)	
**Waist-to-hip ratio**	0.84 ± 0.07	0.77 ± 0.066	t-test, < 0.001
**Duration of hypertension**			–
< 1 year	48 (68.6%)	–	
≥ 1 year	22 (31.4%)	–	
**Family history of hypertension**			–
Yes	47 (67.1%)	–	
No	23 (32.9%)	–	
**Traditional medicine use (past)**			–
Yes	15 (21.4%)	–	
No	55 (78.6%)	–	
**Traditional medicine use (present)**			–
Yes	4 (5.7%)	–	
No	66 (94.3%)	–	
**Blood pressure status**			–
Controlled	26 (37.1%)	–	
Uncontrolled	44 (62.9%)	–	
**Hypertension stage**			–
Stage I	56 (80%)	–	
Stage II	14 (20%)	–	

*Age category was adapted from a previous research article [35].

**Private employee, daily laborer, car driver and non-governmental organization employees; BMI: Body mass index; p-value was calculated by t test for continuous variables and chi-square test for categorical variables; p-values for each cell were calculated from the multiplicity-adjusted residuals for the sex and residence categories.

### Comparison of RDW levels between hypertensive patients and controls

**Fig 2:**
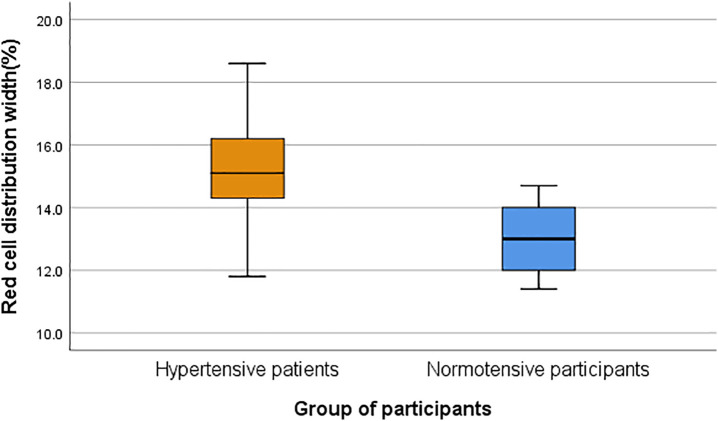
Box-and-whisker plot depicting the distribution of red cell distribution width (RDW) values across study groups at Arba Minch General Hospital, Southern Ethiopia, 2024.

The median red cell distribution width (RDW) was significantly higher in the hypertensive group (15.1%; range, 11.8–18.6%) compared with normotensive controls (13.0%; range, 11.4–14.7%) (p < 0.001), as illustrated below in Table 2 and Fig 2.

**Table 2:** Comparison of RDW levels between hypertensive and normotensive control group at Arba Minch General Hospital, Southern Ethiopia, 2024.

Parameter	Hypertensive (n = 70) Median ± IQR	Normotensive (n = 70) Median ± IQR	P-value
RDW-CV (%)	15.1 ± 2.0%	13.0 ± 2.0%	<0.001

IQR: Interquartile range; RDW-CV: Red cell distribution width coefficient of variation; P-value was calculated by Mann-Whitney u test.

### Comparison of red cell distribution width between stage I and stage II hypertension

The median values of red cell distribution width coefficient variation (RDW-CV) in the stage II hypertensive patients were found to be significantly higher (P < 0.001) as compared to the stage I hypertensive group. The variability of RDW between the two stages showed no significant difference (p = 0.523) (Table 3).

**Table 3:** Comparison of RDW values in stage I and stage II hypertensive patients at Arba Minch General Hospital, Southern Ethiopia, 2024.

Parameter	Stage I HTN (n = 56) Median ± IQR	Stage II HTN (n = 14) Median ± IQR	P-value	
			Mann-Whitney u test	Levene’s test
RDW-CV (%)	14.95 ± 2.10%	16.35 ± 2.60%	<0.001	0.523

HTN: Hypertension; IQR: Interquartile range; RDW-CV: Red cell distribution width coefficient of variation; P-value was calculated by Mann-Whitney u test and Levene’s test.

### Comparison of red cell distribution width between controlled and poorly controlled blood pressure hypertensive patients

This study showed that the median red cell distribution width (RDW) was significantly higher (P < 0.001) in patients with poorly controlled blood pressure compared with hypertensive patients with controlled blood pressure (Table 4).

**Table 4:** Comparison of RDW between controlled and uncontrolled hypertensive patients at Arba Minch General Hospital, Southern Ethiopia, 2024.

Parameter	Controlled BP (n = 5) Median ± IQR	Uncontrolled BP (n = 65) Median ± IQR	P-value
RDW-CV (%)	14.70 ± 1.20%	16.00 ± 2.10%	<0.001

BP: Blood pressure; IQR: Interquartile range; RDW-CV: Red cell distribution width coefficient of variation; P-value was calculated by Mann-Whitney u test.

### Correlation of red cell width with duration of hypertension in hypertensive individuals

**Fig 3:**
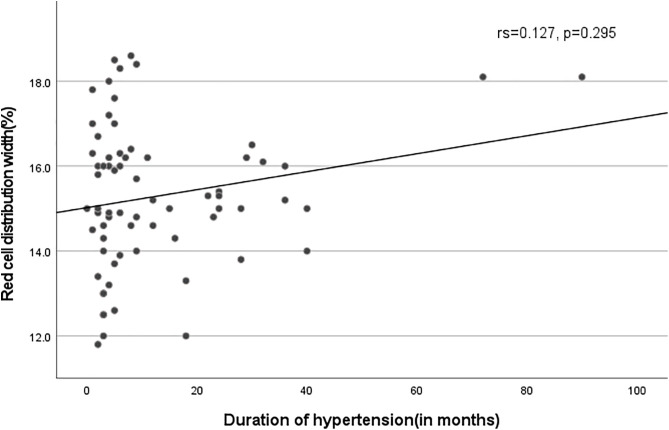
Scatter plot showing the relationship between RDW and duration of hypertension among hypertensive patients at Arba Minch General Hospital, Southern Ethiopia, 2024.

Spearman’s correlation was performed to evaluate the relationship between red cell distribution width (RDW) and duration of hypertension. RDW showed a weak, positive, but statistically non-significant correlation with duration of hypertension among hypertensive patients (rs = 0.127, p = 0.295) (Table 5 and Fig 3).

**Table 5:** Correlation of RDW with duration of illness among hypertensive patients at Arba Minch General Hospital, Southern Ethiopia, 2024.

Parameter	Duration of illness rs(P-value)
RDW-CV (%)	0.127(0.295)

r_s_: spearman’s rank correlation coefficient; RDW-CV: Red cell distribution width coefficient of variation.

### Evaluation of red cell distribution width as a predictor of disease severity in hypertension

**Fig 4:**
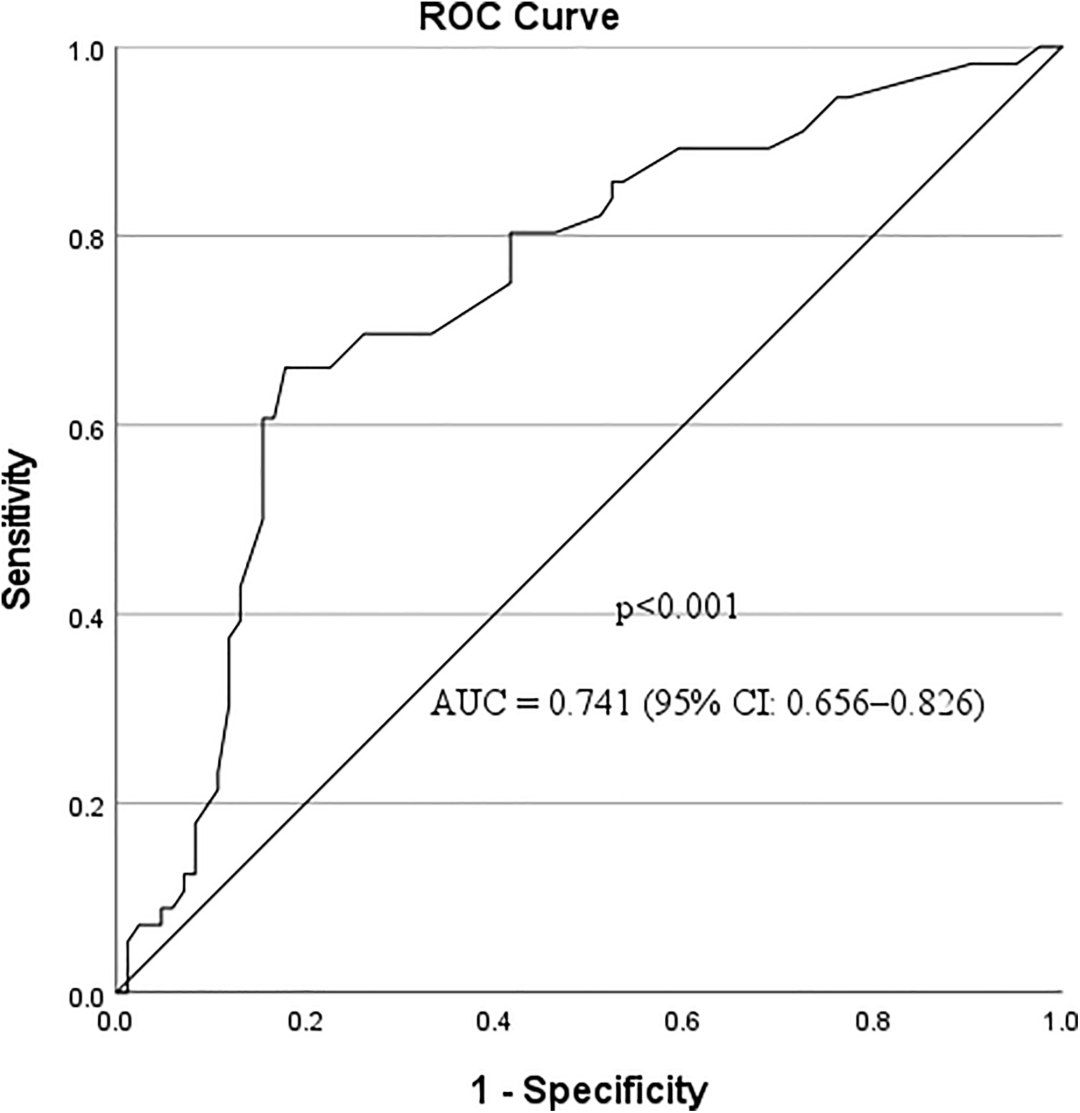
ROC curve for the detection of disease severity among hypertensive patients at Arba Minch General Hospital, Southern Ethiopia, 2024. ROC: Receiver operating characteristic; AUC: Area under the curve; CI: Confidence interval.

The value of RDW-CV for detecting disease severity in hypertensive patients was assessed using receiver operating characteristic (ROC) analysis, which yielded an AUC of 0.741 (95% CI: 0.656–0.826, p < 0.001). At a cut-off value of 14.5%, RDW demonstrated 66.1% sensitivity, 81.4% specificity, 77.9% PPV, 70.4% NPV, and 73.6% overall accuracy (Table 6 and Fig 4).

**Table 6:** Evaluation of RDW for detecting disease severity among hypertensive patients at Arba Minch General Hospital, Southern Ethiopia, 2024.

Cut-off value	Sensitivity, %	Specificity, %	PPV, %	NPV, %	Accuracy, %
RDW-CV (14.5%)	66.1	81.4	77.9	70.4	73.6

RDW-CV: Red cell distribution width coefficient of variation; PPV: Positive predictive value; NPV: Negative predictive value.

### Factors associated with red cell distribution width of study participants

In the multivariable logistic regression analysis, alcohol consumption, khat chewing, C-reactive protein (CRP), and serum creatinine were identified as significant predictors of increased RDW.

Alcohol consumption was significantly associated with higher RDW; participants who consumed alcohol were more likely to have elevated RDW compared to non-consumers (AOR = 6.48; 95% CI: 1.92, 21.85). Similarly, participants who chewed khat were more likely to have increased RDW than non-chewers (AOR = 5.65; 95% CI: 1.06, 29.9). Elevated CRP levels were associated with nearly six-fold higher odds of increased RDW compared to participants with low CRP (AOR = 5.90; 95% CI: 1.67, 20.89). Likewise, higher serum creatinine was independently associated with increased RDW; participants with elevated serum creatinine had 4.35 times higher odds of increased RDW than those with lower levels (AOR = 4.35; 95% CI: 1.19, 15.91). Although age, smoking, fruit and vegetable intake, vitamin supplementation, meat and dairy consumption, salt intake, use of macrocytic medications, and body mass index were significantly associated with RDW in the bivariate analysis, these variables were not significantly associated with RDW after adjustment for potential confounders in the multivariable logistic regression model (Table 7).

**Table 7:** Bivariate and multivariable logistic regression analysis of factors associated with RDW among study participants at Arba Minch General Hospital, Southern Ethiopia, 2024.

Variables	Category	RDW		COR(95%CI)	AOR(95%CI)	P-value
		Normal	Increased			
Age(in years)	18-27	30(34.1%)	11(21.2%)	1	1	
	28-37	23(26.1%)	13(25%)	1.54(0.59, 4.06)	1.08(0.25, 4.73)	0.919
	38-47	19(21.6%)	12(23.1%)	1.72(0.63, 4.68)	0.83(0.18, 3.94)	0.817
	48-57	14(15.9%)	10(19.2%)	1.95(0.67, 5.66)	0.92(0.22, 3.75)	0.904
	≥58	2(2.3%)	6(11.5%)	8.18(1.43, 46.76)	0.65(0.04, 9.78)	0.753
Smoking status	No	79(89.8%)	34(65.4%)	1	1	
	Yes	9(10.2%)	18(34.6%)	4.64(1.89, 11.38)	1.24(0.21, 7.41)	0.817
Alcohol drinking	No	66(75%)	22(42.3%)	1	1	
	Yes	22(25%)	30(57.7%)	4.09(1.97, 8.50)	6.48(1.92, 21.85)	0.003^*^
Khat hewing	No	78(88.6%)	29(55.8%)	1	1	
	Yes	10(11.4%)	23(44.2%)	6.19(2.63,14.56)	5.65(1.06, 29.9)	0.042^*^
Fruit& vegetables	No	3(3.4%)	10(19.2%)	1	1	
	Yes	85(96.6%)	42(80.8%)	0.15(0.04, 0.57)	0.21(0.03, 1.55)	0.125
Vitamin supplement	No	86(97.7%)	48(92.3%)	1	1	
	Yes	2(2.3%)	4(7.7%)	3.58(0.63, 20.29)	4.39(0.34, 57.17)	0.259
Meat&dairy products	No	29(33%)	28(53.8%)	1	1	
	Yes	59(67%)	24(46.2%)	0.42(0.21, 0.85)	1.12(0.33, 3.79)	0.853
Salt use	No	30(34.1%)	28(53.8%)	1	1	
	Yes	58(65.9%)	24(46.2%)	0.44(0.22, 0.89)	0.43(0.13, 1.42)	0.165
Macrocytic drugs use	No	66(75%)	45(86.5%)	1	1	
	Yes	22(25%)	7(13.5%)	0.47(0.184, 1.184)	0.64(0.17, 2.34)	0.495
CRP	≤0.3 mg/dl	73(85.2%)	16(32.7%)	1	1	
	>0.3mg/dl	15(14.8%)	36(67.3%)	10.95(4.87, 24.61)	5.90(1.67, 20.89)	0.006^*^
Creatinine	≤1.2 mg/dl	75(85.2%)	22(42.3%)	1	1	
	>1.2mg/dl	13(14.8%)	30(57.7%)	7.87(3.51, 17.61)	4.35(1.19, 15.91)	0.026^*^
BMI	≤25 kg/m2	46(52.3%)	20(38.5%)	1	1	
	>25 kg/m2	42(47.7%)	32(61.5%)	1.75(0.87, 3.52)	1.33(0.47, 3.75)	0.594

*denotes statistical significance; CRP: C- reactive protein; ≤ 0.3 mg/dl: normal CRP level; > 0.3mg/dl: elevated CRP level; ≤ 1.2 mg/dl: normal serum creatinine; > 1.2mg/dl: elevated serum creatinine; BMI: Body mass index; RDW: Red cell distribution width; COR: Crude odd ratio; AOR: Adjusted odd ratio; CI: Confidence interval.

## Discussion

The present study found that RDW was elevated among hypertensive participants (15.1 ± 2.0%) compared to normotensive controls (13 ± 2.0%), consistent with previous studies by Tanindi A. *et al.* [27] and Chen Y.*et al.* [46]. Increased RDW may reflect ineffective erythropoiesis driven by chronic inflammation [47]. Inflammatory cytokines, including interleukin (IL)-1β, IL-6, and tumor necrosis factor (TNF)-α, may inhibit renal transcription of the EPO gene or impair red blood cell maturation in the bone marrow, allowing immature red cells to enter circulation and increase size variability [48]. In addition to inflammation, renal perfusion and the Renin-Angiotensin-Aldosterone System (RAAS) may also contribute to RDW variability. Reduced renal perfusion stimulates EPO production, while RAAS activation modulates kidney oxygenation and blood pressure. Dysregulation of these pathways in hypertension can result in the release of heterogeneous red blood cells, further increasing RDW [49,50]. The finding of this study support and expand on the concept that RDW has a complex relationship with cardiovascular conditions, including hypertension, contributing to a better understanding of the underlying pathophysiological mechanisms associated with cardiovascular-illnesses. Recognizing the link between RDW and hypertension risk provides clinicians with a novel marker for early detection and treatment of hypertension. Routine blood testing such as RDW measurement can now be used as a risk indicator in hypertensive patients. However, our results contrast with those of Emamain M. *et al* [32]. These discrepancies may reflect differences in study design, population characteristics, hypertension severity, measurement methods, or unmeasured renal and RAAS factors. Future studies incorporating direct measurements of renal perfusion, EPO, and RAAS biomarkers are warranted to clarify these mechanisms and resolve discrepancies.

The present study also demonstrated that the median RDW value was significantly higher in stage II hypertensive patients (16.35 ± 2.60%) compared to stage I patients (14.95 ± 2.10%). This elevation may reflect an intensified inflammatory state associated with increased blood pressure [51]. Chronic hypertension activates various immune pathways, including the NLRP3 inflammasome, leading to the release of pro-inflammatory cytokines such as IL-17, IL-18, and TNF-α. These cytokines can promote vascular smooth muscle cell proliferation and endothelial dysfunction, contributing to arterial stiffness and increased blood pressure [52]. Additionally, oxidative stress resulting from endothelial injury further exacerbates vascular inflammation and blood pressure elevation [53]. Consequently, higher RDW levels could serve as a surrogate marker for the extent of vascular inflammation and endothelial damage in hypertensive patients. These findings suggest that elevated RDW levels are associated with a higher risk of cardiovascular events, with greater elevations reflecting increased disease severity.

In the present study, hypertensive patients with poorly controlled blood pressure had significantly elevated RDW values compared to those with well-controlled blood pressure. This finding is consistent with a study conducted in Harar, Ethiopia [35]. The elevated RDW may result from pronounced inflammation associated with poorly controlled blood pressure, which can impair bone marrow function and release immature erythrocytes, thereby increasing RDW levels [35]. Uncontrolled hypertension also contributes to vascular stiffness and tissue hypoxia, which reduce oxygen delivery to peripheral tissues and stimulate the bone marrow to accelerate erythropoiesis, resulting in the premature release of heterogeneous red blood cells [54]. In addition, heightened sympathetic nervous system activity associated with poorly controlled blood pressure can alter hematopoietic signaling, further disrupting red blood cell maturation and release [55]. These findings suggest that elevated RDW reflects both the inflammatory burden and vascular damage in hypertensive patients, providing a rationale for targeted pharmacotherapy, particularly in those with poorly controlled blood pressure. Since hypertensive individuals might be in more prominent cardiovascular risk than recently recognized, designs focused to lower RDW levels could potentially deliver greater clinical advantages.

This study indicated that duration of hypertension was positively correlated with RDW. This association may be explained by progressive vascular inflammation in patients with longer disease duration. Endothelial damage with subsequent oxidative stress accelerates erythrocyte senescence and erythrophagocytic clearance, resulting in the release of juvenile red cells into peripheral circulation and raises RDW [56]. Prolonged hypertension also promotes chronic activation of inflammatory cytokines which impair erythropoiesis and alter iron metabolism, contributing to greater anisocytosis [57]. In addition, sustained bone marrow stress in long-standing hypertension may drive the premature release of juvenile erythrocytes, while impaired erythropoietin signaling under inflammatory conditions further disrupts normal red cell maturation [58]. The findings implies that an elevated RDW may serve as a predictor of adverse outcomes in patients with chronic hypertension; the poorer prognosis with the longer duration. This provides further insights into the interplay between ineffective erythropoiesis, chronic inflammation, and vascular injury in hypertensive patients.

RDW-CV demonstrated a moderate ability to detect disease severity in hypertensive patients, with an AUC of 0.741 (95% CI: 0.656–0.826, p < 0.001). At a cutoff of 14.5%, RDW showed 66.1% sensitivity, 81.4% specificity, 77.9% PPV, 70.4% NPV, and 73.6% overall accuracy. This finding is consistent with previous studies indicating RDW as a marker of cardiovascular and systemic disease severity [59,60] and supports its role as a complementary marker alongside other clinical and laboratory parameters. Being simple, affordable, and widely available, RDW may be particularly useful in resource-limited settings for patient risk assessment and monitoring. Future studies with larger cohorts and prospective designs are needed to validate these findings.

After adjustment of confounders, through multivariable logistic regression, alcohol drinking, khat chewing, C-reactive protein and serum creatinine were found to be possible predictor variables of RDW. Alcohol consumption was significantly associated with RDW (AOR = 6.48; 95% CI: 1.92, 21.85), consistent with findings reported by Waris S. *et al* [39]. This association may be explained by alcohol metabolites, such as acetaldehyde, which generate free radicals that disrupt red blood cell growth, decrease oxygen-carrying capacity, and shorten erythrocyte lifespan. These oxidative effects, combined with alcohol-induced bone marrow toxicity, impair erythropoiesis and increase red blood cell size variability. Additionally, chronic alcohol intake can lead to nutritional deficiencies, particularly of folate and vitamin B12, further contributing to ineffective erythropoiesis and elevated RDW [39,61]. These results suggest that elevated RDW may serve as a useful biomarker of mortality in critically ill alcoholics and could aid in assessment of potential risks and allows for adjusted treatment choices in these patients. It further gives a more reliable narration of the potential association between chronic alcoholism and systemic inflammation.

In this study, khat chewing was found to be significantly associated with increased RDW (AOR = 5.65; 95% CI: 1.06, 29.9), consistent with a study conducted in Saudi Arabia [62]. This association may be explained by the presence of cathin, the principal psychoactive compound in khat, which is structurally and pharmacologically related to amphetamines. Evidence from studies on other amphetamines—both therapeutic and illicit—shows effects on kidney perfusion, vascular complications, hematopoiesis, and even erythrocyte morphology [63–67]. The other potential mechanism may involve cathin induced hemolysis and ineffective erythropoiesis [62]. Such mechanisms may provide a plausible biological pathway through which khat contributes to alterations in erythrocyte indices. The findings highlight the hematological changes produced by khat. This enhances our understanding of the possible effect of the Catha edulis plant on hematopoiesis, which could result in defective erythropoiesis, thus reflecting the potential of hematological parameters such as RDW to indicate detrimental outcomes among khat chewers. Considering khat within the broader context of amphetamine-related hematological effects, our findings emphasize the need for further mechanistic and clinical research to clarify how stimulant use may influence RDW and hypertension risk.

In this study, C-reactive protein showed significant association with RDW (AOR = 5.90; 95% CI: 1.67, 20.89), which aligns with the findings of Karagoz Ya. *et al* [68]. Several biological mechanisms may explain this relationship. The IL-6/CRP inflammatory axis stimulates hepatic hepcidin production, leading to functional iron deficiency, impaired erythropoiesis, and increased red cell anisocytosis, which raises RDW [69,70]. Furthermore, CRP-related inflammation has been shown to promote red cell aggregation and alter membrane deformability, contributing to greater variability in red cell size [71]. Systemic inflammation and oxidative stress further shorten red cell lifespan and trigger the release of larger, immature erythrocytes into circulation, thereby elevating RDW [24]. Collectively, these mechanisms support the overall idea that the inflammation was one of the main underlying mechanisms responsible for the elevated RDW levels. However, in contrast to our findings, another study showed no correlation between CRP and RDW [72]. This inconsistency may reflect differences in study design, participant characteristics, the short half-life of CRP versus the longer-term changes reflected by RDW, or incomplete adjustment for confounding factors such as iron and micronutrient status.

Serum creatinine was significantly associated with RDW (AOR = 4.35; 95% CI: 1.19, 15.91). In line with this study, another study done in Greece, reported significant association between serum creatinine and RDW [73]. This could be explained by reduced kidney function, which can impair erythropoietin (EPO) production, leading to ineffective erythropoiesis and increased red blood cell size variability [74]. Chronic kidney injury is also associated with systemic inflammation, oxidative stress, and the accumulation of uremic toxins, all of which can shorten red blood cell lifespan, disrupt maturation, and promote the release of immature erythrocytes into circulation, further elevating RDW [75] Another possible mechanism is the effect of intensive antihypertensive therapy, which may lower renal perfusion pressure, reduce glomerular filtration rate without functional nephron loss, and consequently impair EPO [76]. These pathways suggest that renal dysfunction directly and indirectly influences red cell homeostasis, contributing to higher RDW levels in hypertensive patients. The finding also indicates that changes in RDW could reflect therapeutic effects, highlighting that RDW may serve as an accurate risk stratification tool to help clinicians optimize treatment and ultimately improve clinical outcomes.

In this study, age, BMI, and smoking were not significantly associated with RDW, although some previous studies have reported associations [40,77,78]. These differences may reflect variations in study populations, socio-demographic characteristics, genetic and biological factors, measurement methods, lifestyle or environmental influences.

In our study, we observed considerable rise of RDW values among patients compared to controls. RDW is cost-effective and readily available measure, as it is routinely reported in standard complete blood cell counts. Although blood pressure measurement remains the primary and most reliable method for diagnosing hypertension—being more direct, rapid, and less influenced by confounding factors—our findings suggest that RDW may serve as a complementary prognostic marker in the clinical risk assessment of hypertensive patients. Elevated RDW levels may reflect disease severity or suboptimal treatment adherence, and monitoring RDW could aid in risk stratification and clinical management of hypertension. Thus, RDW can serve as a supportive marker to help clinicians identify patients at higher risk and optimize individualized care, rather than as a standalone diagnostic tool.

Our findings show that RDW increased with advancing hypertension stage, highlighting its potential role as a novel biomarker beyond conventional CBC parameters. Unlike CBC, which provides descriptive hematological indices, RDW reflects anisocytosis and integrates the impact of inflammation, oxidative stress, and vascular dysfunction—all of which are relevant to the pathophysiology of hypertension. These findings suggest that RDW has prognostic value in risk evaluation and disease monitoring, offering clinicians an accessible and cost-effective tool for improving hypertension management.

### Strengths and limitations

Strengths of this study include detailed information on clinical and laboratory test parameters. Our study adds evidence on RDW among hypertensive patients in the Ethiopian population, particularly in Southern Ethiopia, which could support further related research and offer health care practitioners a cheap, readily available tool for evaluating hypertensive patients. However, this study has certain limitations to be addressed such as being cross-sectional nature of the study didn’t establish causal relationship. Besides, we utilized a single hematology analyzer machine (Mindray) without intermachine comparisons. Markers of oxidative stress and lipid profiles were not assessed. Moreover, we lacked serum iron data, an essential factor that could affect our results. This study used a 1:1 case-to-control ratio, but including two or more controls per case in future studies could improve representativeness and strengthen generalizability. ANOVA, post hoc analysis and interaction testing were not performed, which may limit detection of subgroup differences and the strength of observed associations. Finally, we did not perform multiple linear regressions to evaluate the relationship between RDW and continuous blood pressure measurements while adjusting for pharmaceutical control and other potential confounders. Therefore, more advanced studies with larger and more heterogeneous populations are required to address these issues and to further elucidate the role of RDW in the progression and management of hypertension-related complications.

## Conclusion

The present findings indicate that RDW levels were significantly higher in hypertensive cases compared to normotensive controls. Among hypertensive patients, those with stage II disease exhibited significantly greater RDW values than those with stage I hypertension. Similarly, patients with poorly controlled blood pressure had higher RDW levels compared to those with controlled blood pressure. A weak positive correlation was observed between RDW and the duration of hypertension. RDW has been shown to have moderate predictive power for detecting disease severity in hypertensive patients. Furthermore, elevated RDW was independently associated with alcohol consumption, khat chewing, C-reactive protein, and serum creatinine. These findings suggest that RDW could serve as an important adjunct marker for clinicians and public health experts. Given its cost-effectiveness, wide availability, and prognostic value, RDW should be considered in the assessment and follow-up of hypertensive patients, rather than relying solely on conventional CBC parameters. Incorporating RDW into routine assessment may enhance early detection of complications, improve risk stratification, and support more personalized care and monitoring of hypertensive patients.

## Supporting information

S1 File Annex. Questionnaire for study participants.
(PDF)

S2 File Annex. SPSS data sheet.
(CSV)

S3 File Annex. Validation and reliability testing of the study questionnaire.
(PDF)

## Abbreviations

BMI: Body mass index
BP: Blood pressure
CBC: Complete blood cell count
CRP: C-reactive protein
DBP: Diastolic blood pressure
EDTA: Ethylene diamine tetra-acetate
EPO: Erythropoietin
HTN: Hypertension
RDW: Red cell distribution width
SBP: Systolic blood pressure
SST: Serum separator tube
WHR: Waist to hip ratio
