# Supplementary material for: Red cell distribution width and associated factors among hypertensive patients attending Arba Minch General Hospital, Southern Ethiopia: A comparative cross-sectional study
Source: PLoS One. 2025 Nov 13;20(11):e0336409. doi: 10.1371/journal.pone.0336409 (PMC12614529; doi:10.1371/journal.pone.0336409)
Supplement: S1 File — Questionnaire for study participants. (PDF) [file pone.0336409.s001.pdf]

Annex : Questionnaire in English version

ARBA MINCH UNIVERSITY COLLEGE OF MEDICINE AND HEALTH SCIENCES
DEPARTMENT OF BIOMEDICAL SCIENCES

A questionnaire is developed to assess the red cell distribution width and associated factors among hypertensive patients attending Arba Minch General Hospital. A Comparative Cross-Sectional Study

Code_________________

Part I: Socio-demographic characteristics

No	Variables	Response
1	Age(year)	___________
2	Sex	1. Male 2. Female
3	Marital status	1. Single 2. Married 3. Divorced 4. Widowed
4	Educational status	1. No education 2. Primary 3. Secondary 4. College/university and above
5	Residence	1. Rural 2. Urban
6	Occupation	1. Farmer 2. Merchant 3. Government employee 4. Others
7	Monthly income(in birr)	______________

Part II: Behavioral factors and Nutrition

1	Do you smoke?	1. Yes 2. No
2	If yes, how many years ago did you start smoking?	______________
3	How many times do you smoke?	1. Daily 2. Frequently 3. Rarely
4	On average, how many times do you smoke each day?	_______________
5	Do you drink any alcohol such as beer, Tella, Bordie, Tej, Arake, wine?	1. Yes 2. No
6	How many times do you drink alcohol?	1. Daily 2. Frequently 3. Rarely
7	Did you chew chat?	1. Yes 2. No
8	If yes, when did you start chewing chat?	_______________
9	Do you use fruit and vegetables?	1. Yes 2. No
10	How often do you use fruit and vegetables per week?	1. Not at all 2. 1-3 days per week 3. 4-7 days per week
11	Are you taking any vitamin or nutritional supplements?	1. Yes 2. No
12	If yes, specify	_________________
13	Do you use meat or dairy products?	1. Yes 2. No

14	How often do you use meat and dairy products?	1. Daily 2.Frequently 3.Rarely
15	Do you add salt to your food?	1. Yes 2. No
16	How often do you add salt to your food?	1. Daily 2.Frequently 3.Rarely
17	Have you ever used any of the following medications?<br>1. Sulfa drugs 2. Antiretrovirals<br>2. Anticonvulsants 4. Chemotherpy	1. Yes 2. No

Part III: History of hypertension (for case group only)

1	How long have you had hypertension?	________
2	Do you have family history of hypertension?	1. Yes 2.No
3	Have you ever seen a traditional healer for treatment of hypertension or other diseases?	1. Yes 2. No
4	Are you currently seeing a traditional healer for treatment of hypertension or other diseases?	1. Yes 2.No
5	Are you currently using drugs for blood pressure control?	1. Yes 2. No

Part IV: Anthropometric measurement

Height ____cm Weight ____kg BMI ____kg/m2 WC ____cm HC___cm WHR___SBP _____mmHg DBP___mmHg

Part V: Laboratory test parameters

1. Red cell distribution width ___________%
2. Mean corpuscular volume ___________fl
3. Serum creatinine ___________________mg/dL
4. Serum albumin ____________________g/dL
5. C-reactive protein ___________________mg/dL

Thank you for your participation.
