# Supplementary material for: Red cell distribution width and associated factors among hypertensive patients attending Arba Minch General Hospital, Southern Ethiopia: A comparative cross-sectional study
Source: PLoS One. 2025 Nov 13;20(11):e0336409. doi: 10.1371/journal.pone.0336409 (PMC12614529; doi:10.1371/journal.pone.0336409)
Supplement: S3 File — Validation and reliability testing of the study questionnaire. (PDF) [file pone.0336409.s003.pdf]

Validation and Reliability of the Study Questionnaire

Section	Number of Items	Content Validity (Expert Review)	Cronbach's Alpha (Reliability)	Notes
Socio-demographics	7	Reviewed by 3 experts; all items deemed relevant	0.78	Pretested on 5% of participants outside main study
Lifestyle factors (smoking, alcohol, khat, diet)	17	Reviewed by 3 experts; minor wording adjustments made	0.82	Pretested on 5% of participants outside main study
Clinical history & medication use	5	Reviewed by 3 experts; all items relevant	0.76	Pretested on 5% of participants outside main study
Anthropometric parameters	8	Reviewed by 3 experts; all items relevant	0.80	Pretested on 5% of participants outside main study
Laboratory sample collection	5	Reviewed by 3 experts; all items relevant	0.84	Pretested on 5% of participants outside main study
Overall questionnaire	29	Average CVI = 0.90	0.80	Back-translation confirmed accuracy
